# Supplementary material for: Identification of Pre-Diabetic Biomarkers in the Progression of Diabetes Mellitus
Source: Biomedicines. 2021 Dec 30;10(1):72. doi: 10.3390/biomedicines10010072 (PMC8773205; doi:10.3390/biomedicines10010072)
Supplement: Supplementary file 1 [file biomedicines-10-00072-s001.zip › biomedicines-1522807-Supplementary.pdf]

Figure S1: Selected pre-diabetes bio-marker expression levels in the liver of *ob/ob* and *db/db* mice.

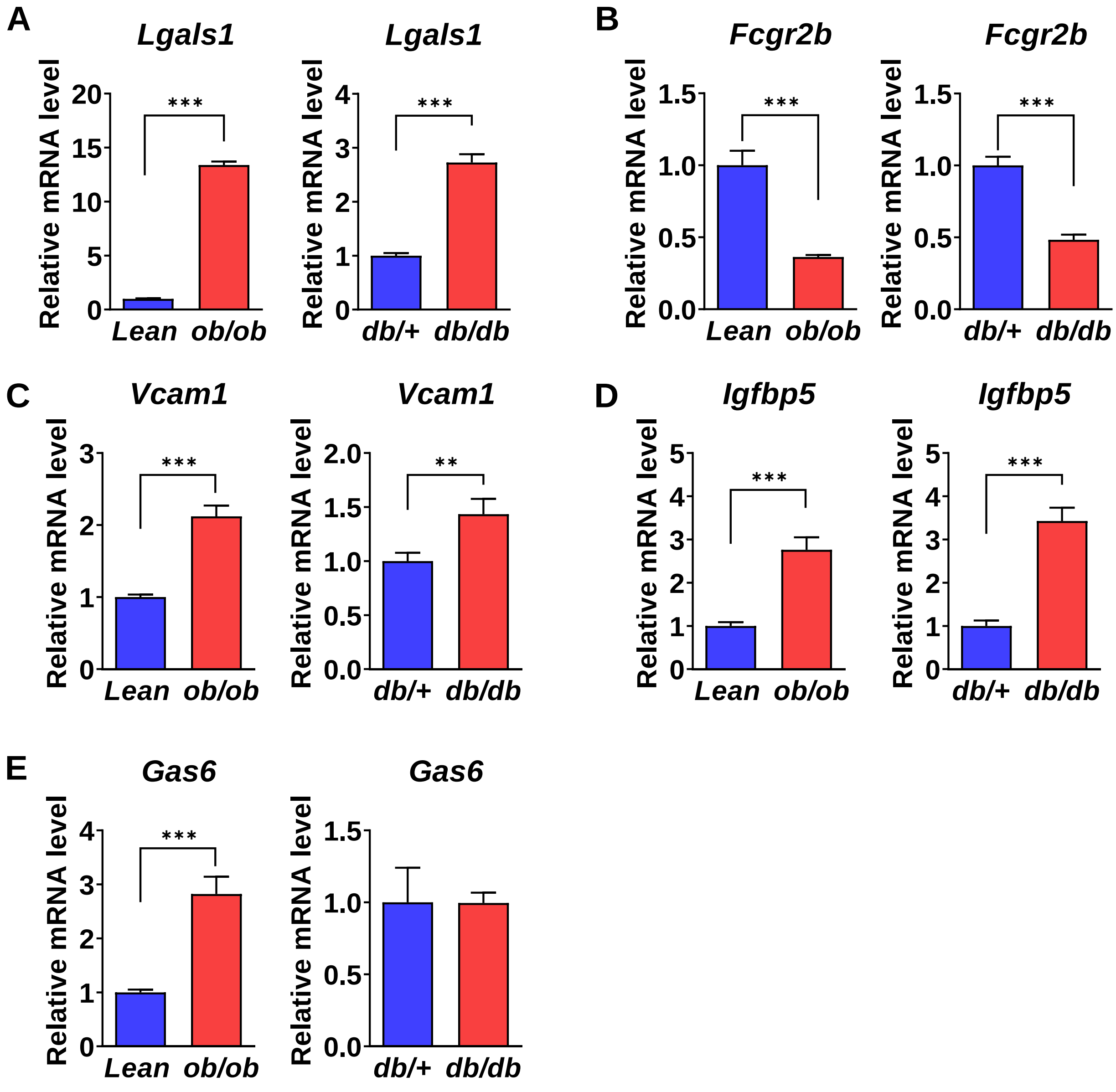

Figure S1: Expression levels of the selected pre-DM bio-markers in the liver samples of *ob/ob* and *db/db* mice. (A-E) mRNA expression of *Lgals1*, *Fcgr2b*, *Vcam1*, *Igfbp5* and *Gas6*. (n=5). **p < 0.01 and ***p < 0.001 *Lean* vs.*ob/ob* or *db/+* vs *db/db*.

Figure S2: Diabetes marker gene expression levels in the liver of *ob/ob* and *db/db* mice.

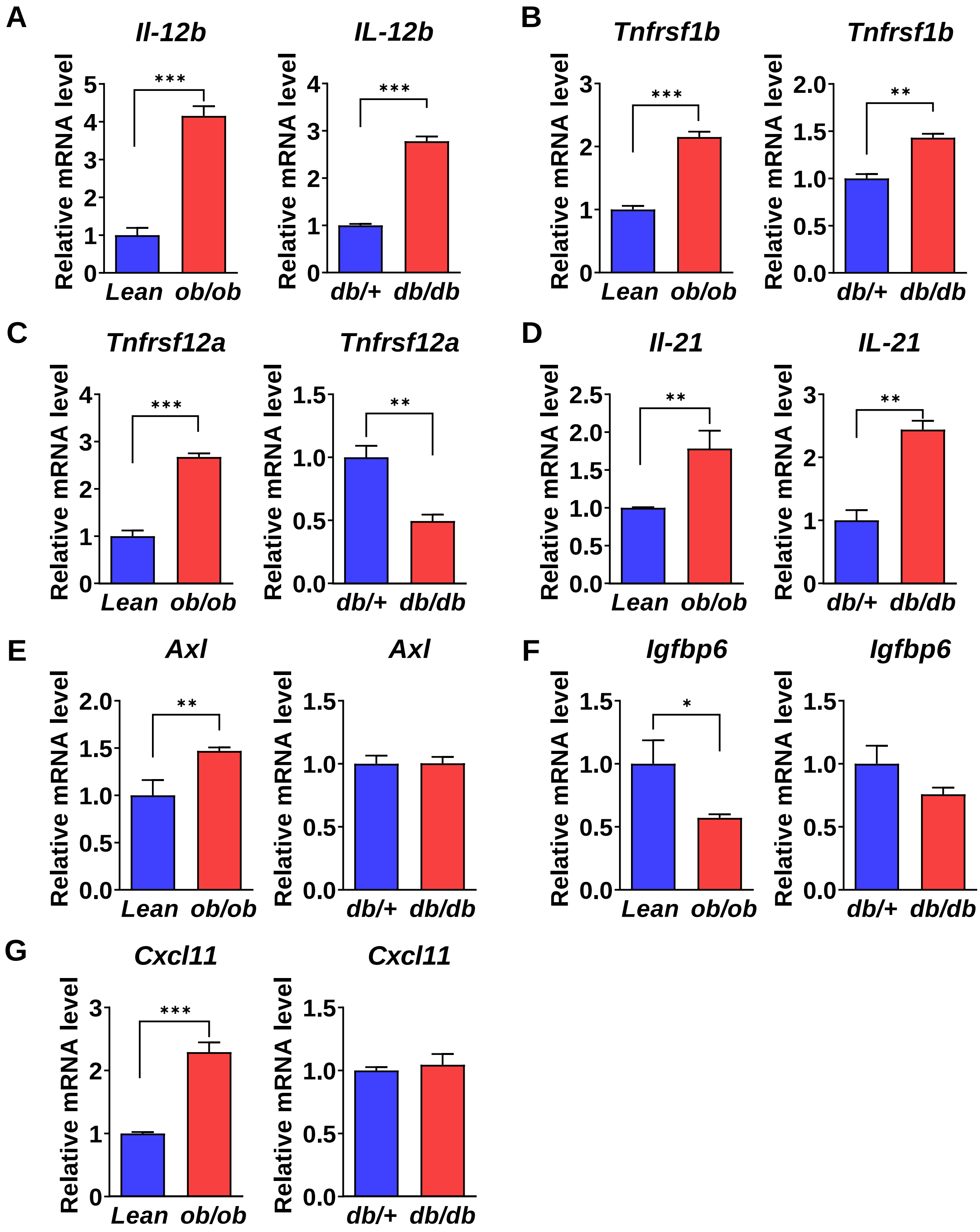

Figure S2: Expression levels of the diabetes markers in the liver samples of *ob/ob* and *db/db* mice. (A-G) mRNA expression of *Il-12b*, *Tnfrsf1b*, *Tnfrsf12a*, *Il-21*, *Axl*, *Igfbp6* and *Cxcl11*. (n=5). **$p < 0.01$ and ***$p < 0.001$ *Lean* vs.*ob/ob* or *db/+* vs *db/db*.
